# Chronic Social Defeat During Adolescence Induces Short- and Long-Term Behavioral and Neuroendocrine Effects in Male Swiss-Webster Mice

**DOI:** 10.3389/fnbeh.2021.734054

**Published:** 2021-09-30

**Authors:** Héctor Miguel Mancha-Gutiérrez, Erika Estrada-Camarena, Lilian Mayagoitia-Novales, Elena López-Pacheco, Carolina López-Rubalcava

**Affiliations:** ^1^Departamento de Farmacobiología, CINVESTAV-Sede Sur Coapa, Mexico City, Mexico; ^2^Laboratorio de Neuropsicofarmacología, Dirección de Neurociencias, Instituto Nacional de Psiquiatría Ramón de la Fuente Muñiz, Mexico City, Mexico; ^3^Departamento de Etologia, Dirección de Neurociencias, Instituto Nacional de Psiquiatría Ramón de la Fuente Muñiz, Mexico City, Mexico

**Keywords:** adolescence, social defeat, stress, depression, cognition, testosterone, corticosterone, serotonin transporter

## Abstract

Chronic stress exposure during adolescence is a significant risk factor for the development of depression. Chronic social defeat (CSD) in rodents is an animal model of depression with excellent ethological, predictive, discriminative, and face validity. Because the CSD model has not been thoroughly examined as a model of stress-induced depression within the adolescence stage, the present study analyzed the short- and long-term behavioral and neuroendocrine effects of CSD during early adolescence. Therefore, adolescent male Swiss-Webster (SW) mice were exposed to the CSD model from postnatal day (PND) 28 to PND37. Twenty-four hours (mid-adolescence) or 4 weeks (early adulthood) later, mice were tested in two models of depression; the social interaction test (SIT) and forced swimming test (FST); cognitive deficits were evaluated in the Barnes maze (BM). Finally, corticosterone and testosterone content was measured before, during, and after CSD exposure, and serotonin transporter (SERT) autoradiography was studied after CSD in adolescent and adult mice. CSD during early adolescence induced enduring depression-like behaviors as inferred from increased social avoidance and immobility behavior in the SIT and FST, respectively, which correlated in an age-dependent manner with SERT binding in the hippocampus; CSD during early adolescence also induced long-lasting learning and memory impairments in the Barnes maze (BM). Finally, CSD during early adolescence increased serum corticosterone levels in mid-adolescence and early adulthood and delayed the expected increase in serum testosterone levels observed at this age. In conclusion: (1) CSD during early adolescence induced long-lasting depression-like behaviors, (2) sensitivity of SERT density during normal brain development was revealed, (3) CSD during early adolescence induced enduring cognitive deficits, and (4) results highlight the vulnerability of the adolescent brain to social stressors on the adrenal and gonadal axes, which emphasizes the importance of an adequate interaction between both axes during adolescence for normal development of brain and behavior.

## Introduction

During adolescence, chronic stress exposure can increase the susceptibility to develop various psychiatric disorders, such as depression (Lupien et al., 2009). Depression is the most common psychiatric disorder characterized by depressed mood, anhedonia, irritability, diminished ability to think or concentrate, disturbances in appetite and sleep, and recurrent thoughts of death (American Psychiatric Association, 2013). On the other hand, adolescence is the transition stage between childhood and adulthood, which is characterized by significant changes at hormonal, somatic, and behavioral levels, as well as a continuous neurodevelopmental process, which collectively lead to reproductive efficiency and social and cognitive maturation of the individual (Lupien et al., 2009; Holder and Blaustein, 2014). It has been shown that different forms of social stress during adolescence may result in long-lasting physiological, behavioral, and neuronal consequences, which are associated with depressive symptoms in the same adolescence stage or may even persist into adulthood (Buwalda et al., 2011). Bullying is an ethologically relevant social stressor prevalent among adolescents (Xu et al., 2016). Worldwide, between 10 and 30% of children and adolescents are regularly involved in school bullying (Resende et al., 2016). Therefore, bullying during adolescence has become a significant risk factor for several psychiatric disorders, such as depression (Iñiguez et al., 2016; Resende et al., 2016).

Chronic social defeat (CSD) is an animal model of depression with excellent ethological, predictive, discriminative, and face validity (Golden et al., 2011), which uses a naturalistic stressor as opposed to other experimental approaches that incorporate other environmental stressors (Iñiguez et al., 2016). CSD in mice has been extrapolated to bullying in humans, with the aggressor mouse being equivalent to the bully and the defeated mouse being equivalent to the victim (Huang et al., 2013; Shimizu et al., 2020). In adult male mice, the CSD model can induce depression-like behaviors such as social avoidance (Golden et al., 2011), behavioral despair (Wang et al., 2019), anhedonia (Shu and Xu, 2017), cognitive deficits such as memory impairments (Martin et al., 2017), physiological disturbances like hyperreactivity of the hypothalamus-pituitary-adrenal axis (Keeney et al., 2006), and weight loss (Krishnan et al., 2007). Although CSD is perhaps a valid animal model for “bullying,” most studies with this protocol have been made in adult mice; thus, it is equally important to examine how the adolescent brain responds to social stress (Resende et al., 2016). Here, the CSD protocol is further examined as a model of early experiential or stress-induced depression during the adolescent stage (Iñiguez et al., 2014).

It is known that disturbances in serotonergic neurotransmission are involved in a wide range of psychiatric disorders, such as depression. Evidence suggests an interaction between the serotonin transporter (SERT) and stress. For example, adult male rats exposed to social defeat upregulated SERT protein levels in the dorsal raphe nuclei (DRN); this effect was mimicked by oral corticosterone ingestion and was prevented by treatment with mifepristone and spironolactone (Zhang et al., 2012). Conversely, social defeat in adult male mice down-regulated the mRNA levels of the SERT gene in the raphe nuclei area (Boyarskikh et al., 2013). Even though these results might seem contradictory, they highlight SERT’s susceptibility to stressors and evidence the need to study the SERT-stress interaction. Recently, Reisinger et al. (2019) first demonstrated a quantitative *in vivo* evaluation of SERT density in the mouse brain by positron emission tomography (PET). These authors found that chronic corticosterone treatment decreased SERT density in the hippocampus, striatum, thalamus, and cerebral cortex of adult male mice and induced the development of a behavioral phenotype associated with depression, evidencing the involvement of SERT in the neurobiology of stress-induced depression (Reisinger et al., 2019).

Moreover, a significant percentage of people suffering from stress-induced psychiatric disorders also experiment cognitive deficits (Blanco-Gandia et al., 2019). For example, clinical depression is frequently associated with cognitive impairments, which can be directly related to the number of depressive episodes previously experienced, and can also be present at the onset of other depressive symptoms (Miskowiak et al., 2016). Accordingly, cognitive deficits are increasingly recognized as an independent and significant risk factor for the development of depression (Xu et al., 2018). We aim to provide further insight regarding how CSD during adolescence interferes with cognitive performance (Huang et al., 2013) during the time of adolescence as well as later on in adulthood.

Finally, the hormonal changes in adolescence are related to both the hypothalamic-pituitary-adrenal (HPA) and the hypothalamic-pituitary-gonadal (HPG) axes. The HPA axis is responsible for orchestrating the stress response, and its activation results in glucocorticoid synthesis and release from the adrenal glands, which promotes the re-establishment and maintenance of homeostasis (De Kloet et al., 2005; Godoy et al., 2018). At the same time, the HPG axis is responsible for reproductive competence since it promotes growth and maturation of gametes and steroid hormones synthesis from the gonads, estradiol in females, and testosterone in males (Oyola and Handa, 2017). Interestingly, adolescents (both females and males) and adult females exhibit a more robust and more extended stress response to stressors compared to adult males (Romeo, 2010; Oyola and Handa, 2017; Brivio et al., 2020). It has been proposed that this difference in the stress response could be secondary to a general suppression of the HPA axis activity due to the increase in serum testosterone, regulated by HPG axis activity, experienced by males during adolescence (Bale and Epperson, 2015; Brivio et al., 2020). Hence, the CSD effects on the activity of the HPG axis would benefit from further research.

The present study aimed to evaluate the short- (mid-adolescence) and long-term (early adulthood) effects of the CSD model during early adolescence on behaviors like depression, the SERT density in the prefrontal cortex (PFC) and hippocampus, the visuospatial learning and memory, and the serum levels of corticosterone and testosterone, in male mice of the Swiss Webster (SW) strain.

## Materials and Methods

### Animals

We used adolescent male SW mice as study subjects and adult male SW mice as aggressors, provided by our breeding facilities at postnatal day (PND) 21 for adolescents or after PND70 for adults. Adolescent mice were group-housed (4–6 per cage) until the beginning of experiments (PND28). Adult mice were individually housed for 1 week and later screened for aggressive behavior and selected as aggressor mice in the protocol, based on the behavioral features described by Golden et al. (2011). All mice had access to tap water and standard chow *ad libitum* and were kept in a temperature-controlled room (21–23°C) with an inverted light/dark cycle (12 h/12 h, lights on starting at 21:00 h). Our Institutional Ethics Committee (CICUAL Number of Project 379-02) approved all experimental procedures and followed regulations established by the Mexican Official Norm (NOM-062-ZOO-1999) for the use and care of laboratory animals.

### Chronic Social Defeat Model

We used the CSD model described by Golden et al. (2011). Briefly, an adolescent mouse (PND28) was placed directly within the aggressor mouse cage compartment for 5 min; at the end of the 5 min of social defeat, the adolescent mouse was separated from the aggressor mouse by a clear perforated acrylic division that allowed to maintain sensory contact with the aggressor mouse for a 24-h period. This procedure was replicated for 10 consecutive days, and the adolescent mice were rotated with a new aggressor every day along the 10 days of social defeat to avoid habituation to a single aggressor. On the other hand, control mice were pair-housed with other adolescent mice but kept on the opposite side of the cage separated by the acrylic divider, all control mice were rotated every day likewise to defeated mice, but they were never allowed physical contact with their respective cage mate.

### Behavioral Tests

#### Social Interaction Test

We used the SIT to assess the social avoidance behavior, as described previously by Berton et al. (2006) and Golden et al. (2011). Briefly, the SIT consisted of two sessions conducted under red light conditions. In the first 2.5-min session (social target absent), a control or defeated mouse was allowed to explore freely an open field arena (42 cm length × 42 cm width × 42 cm height) equipped with a wire-mesh enclosure (10 cm length × 10 cm width × 6.5 cm height) on one side of the arena, which remained empty during the first session. At the end of the first session, the control or defeated mouse was removed from the open field arena, and an unfamiliar SW mouse of the same age as the experimental subject (social target) was placed into the wire-mesh enclosure. In the second 2.5-min session (social target present), the control or defeated mouse was reintroduced into the arena containing the social target within the wire-mesh enclosure and was allowed to explore freely the open field arena again. The SIT was videotaped and analyzed with the Top Scan View 2.0 software (Clever Sys Inc., Reston, VA, United States) to determine the social interaction ratio (SIR). The SIR was obtained by dividing the time (seconds) that the control or defeated mouse spent in the interaction zone (an 8 cm surrounding the wire-mesh enclosure area) when the social target was present by the time spent in the interaction zone when the social target was absent. If the SIR was < 1, it meant that the mouse was susceptible and developed the social avoidance behavior; conversely, if the social interaction ratio was ≥ 1, it meant that the mouse was resilient and did not develop the social avoidance behavior (Golden et al., 2011).

#### Forced Swimming Test

We used the FST to assess the behavioral despair, following the previous description by Castagné et al. (2011). Briefly, the FST was conducted by placing a control or defeated mouse inside individual glass cylinders (24 cm height × 13 cm diameter) filled with 10 cm of water at 23 ± 2°C, for 6 min. Then, the mouse was retired from the cylinders, dried with paper towels, placed in heated cages, and, 30 min later, the mouse was returned to its home cage. The FST was videotaped for later scoring by an experienced observer blind to the experimental manipulations. A time sampling technique was employed in which during the last 4 min of the FST, at the end of each 5 s, the behavior presented mostly by the mouse was scored (Detke et al., 1995): (1) Immobility—when the mouse floated in the water without struggling and performed only those necessary movements to keep the head above the water, (2) swimming—when the mouse performed more active movements, moving around the cylinder, and (3) climbing—when the mouse performed vigorous movements with the forepaws in and out of the water, usually against the walls of the cylinder. An increase in immobility behavior, resulting from reducing active behaviors (swimming and climbing), is considered a measure of behavioral despair.

#### Barnes Maze

We decided to use the BM to evaluate visuospatial learning and memory (Paul et al., 2009; Sharma et al., 2010) because it reduces the stress effects on cognitive performance, as it is less stressful and more suitable for mice since these animals exhibit a lower performance in the Morris maze, one of the most used mazes for the evaluation of visuospatial learning and memory (Paul et al., 2009). Our BM consisted of an open circular platform (92 cm in diameter) elevated at a 90-cm height that contains 18 holes (each 9.5 cm in diameter) equally distributed around the platform and separated by 7.5 cm, one of which has a small dark chamber (target box) located underneath (not visible from the platform). The BM has been considered appropriate for mice because of their propensity to find and escape through small holes and the natural preference of rodents for a dark environment (Sharma et al., 2010). During the test, mice are exposed to mild stressors, both light and white noise, to induce the escape behavior, thus, they will try to escape from the open platform surface toward the target box. Furthermore, the maze is in a room that contains visuospatial clues on its walls, which always remain at the same position and serve to orient the mouse about the target box location that remains always in the same position. Briefly, the test was performed under normal light conditions and consisted of three phases (habituation, acquisition, and retention) and lasts 12 days (Sunyer et al., 2007).

Habituation phase (day 1): performed at least 1 h before the acquisition phase, placing a control or defeated mouse at the center of the platform under a crystal glass for 15 s. After that, the white noise source was turned on, and the crystal glass with the mouse inside was carefully driven to the hole over the target box and was subtly incited to enter the target box. The white noise source was turned off, and the mouse was kept inside the target box for 60 s.

Acquisition phase (days 1–4): this phase consisted of two trials each day with a 30 min interval inter-trial, from days 1 to 4 (8 trials in total) (Riedel et al., 2018). In each trial, a control or defeated mouse was placed at the center of the platform under a dark-colored acrylic cylinder, allowing the mouse to be in random orientation before each trial for 15 s; later, the cylinder was lifted, and the mouse was allowed to explore freely on the platform for 3 min. The trial ended when the mouse entered the target box or after 3 min had elapsed. In the latter case, the mouse was subtly placed inside the target box. In both cases, when the mouse was inside the target box, the white noise source was turned off, and the mouse was kept in it for 60 s.

Retention phase (days 5 and 12): this phase consisted of a test trial, performed at 1 day (day 5) or 8 days (day 12) after the last day of the acquisition phase to evaluate the short- or long-term retention, respectively (Sunyer et al., 2007). The test trial was performed like the trials in the acquisition phase, but the test trial lasted 1.5 min. It is important to note that there were no trials between days 5 and 12.

In all trials, the platform was cleaned before each trial began to avoid olfactory clues, and the platform was rotated around its central axis after each trial to control the possible remaining odor clues. All trials were videotaped and analyzed with the Top Scan View 2.0 software (Clever Sys Inc.) to determine the primary latency, defined as the time elapsed between the start of the trial and the first encounter with the hole over the target box.

#### Serum Hormone Immunoassays

We used the Enzo Life Science enzyme-linked immunosorbent assay (ELISA) kits (Farmingdale, NY, United States) to determine the serum content of both corticosterone (ADI-900-097) and testosterone (ADI-900-065). The assays were performed following manufacturer’s instructions, and the plates were read at 405 nm with the Stat Fax 2100 (Fisher Bioblock Scientific, FL, United States). All samples were run in duplicate. For the corticosterone assay, cross-reactivity was achieved with 28.6% deoxycorticosterone, 1.7% progesterone, 0.28% tetrahydrocorticosterone, 0.18% aldosterone, 0.13% testosterone, 0.046% cortisol, and < 0.03% for all other hormones; the inter-assay variability was < 15% and the intra-assay variability was < 10%; the sensitivity of the assay was 26.99 pg/mL. For the testosterone assay, cross-reactivity was reached with 14.64% 19-hydroxytestosterone, 7.2% androstenedione, 0.72% dehydroepiandrosterone, 0.40% estradiol, and < 0.001% for all other hormones; both the inter-assay and the intra-assay variabilities were < 15%, and the sensitivity of the assay was 5.67 pg/ml.

#### Serotonin Transporter Autoradiography

The frozen brains of control and defeated mice were cut in 20-μm coronal slices at −20°C using a Hyrax C25 cryostat (Zeiss, Germany). The coronal brain slices were then mounted on gelatinized slides and stored at −80°C until they were processed by autoradiography, at which time they were brought to room temperature (RT) for at least 2 h. For the autoradiography process, the slides with the coronal brain slices were pre-incubated in Tris-HCl (50 mM, pH 7.7) containing 120 mM NaCl and 5 mM KCl for 30 min at RT. Afterward, the slides were incubated for 60 min at RT with [3H]-paroxetine (0.5 nM; 25 Ci/mM, Perkin Elmer, Shelton, CT, United States), a selective serotonin reuptake inhibitor (SSRI) (Giannaccini et al., 2011; Solich et al., 2011). Non-specific binding was determined in the presence of 30 μM paroxetine hydrochloride (Sigma Aldrich, Toluca, Mexico). Following incubation, slides were washed twice with cold Tris-HCl for 10 min. Finally, the slides were quickly dipped in cold distilled water (5 s) and dried using a cold air device. The slides were placed inside X-ray cassettes with tritium-containing standards (American Radiolabeled Chemicals, St. Louis, MO, United States) to allow the slides to be exposed to tritium-sensitive Kodak film for 6 months. The film was developed with Kodak GBX Developer (Sigma Aldrich, Toluca, Mexico) for 5 min at 20°C. The tritium standards were used to obtain the standard curve of optical density (OD). To measure radioactivity, 10 OD readings per structure were taken in the brain slices, at least five different adjacent slices were read and averaged (Páez-Martínez et al., 2020). OD values were converted to fmol/mg of tissue using video analysis software (Quantity One 1-D). The brain areas evaluated included the PFC and dorsal (DH) and ventral hippocampus (VH), which were identified by the stereotaxic coordinates of Paxinos and Franklin for the mouse brain (Paxinos and Frankiln, 2001).

#### Statistical Analysis

The social interaction ratios, behaviors in the FST, [3H] paroxetine specific bindings, and primary latencies in the retention phase of the BM were analyzed using two-way ANOVA test, the factors being age and stress. The serum corticosterone and testosterone contents were analyzed using two-way ANOVA with repeated measures, the factors being age and stress. The areas under the curve of corticosterone and testosterone were analyzed using student *t*-test. The correlations between SERT binding-depression-like behaviors, serum corticosterone-age of mice, serum testosterone-age of mice, and serum corticosterone-serum testosterone were determined using the Pearson correlation test. The primary latencies in the acquisition phase of the BM were analyzed using a repeated measures ANOVA (RM-ANOVA) test. Significance was set at *p* < 0.05 for all tests. All statistical analyses were conducted using GraphPad Prism (GraphPad Software, San Diego, CA, United States).

#### Experimental Procedures

##### Experiment 1. Short- and Long-Term Depression-Like Behaviors Induced by Chronic Social Defeat During Early Adolescence

Four independent groups of mice (*n* = 7 per group) were exposed to 10 consecutive days of CSD or control manipulation during early adolescence from PND28 to 37. For short-term studies, two groups (one control and one defeated group) were evaluated in mid-adolescence in the SIT at PND39 and the FST at PND40. The other two groups (one control and one defeated group) were group-housed without any manipulation for 4 weeks until reaching early adulthood for long-term studies. These mice were evaluated in the SIT at PND67 and the FST at PND68.

##### Experiment 2. Short- and Long-Term Effects on Serotonin Transporter Binding Induced by Chronic Social Defeat During Early Adolescence

Four independent groups of mice (*n* = 4 per group) were exposed to 10 consecutive days of CSD or control manipulation during early adolescence from PND28 to 37. For the short-term studies, two groups (one control and one defeated group) were euthanized in mid-adolescence by decapitation at PND39. For the long-term studies, two groups (one control and one defeated group) were group-housed without any manipulation for 4 weeks until reaching early adulthood; later, mice were euthanized in early adulthood by decapitation at PND67. In all cases, the mouse brains were quickly removed, frozen on dry ice, and stored at −80°C until use.

##### Experiment 3. Short- and Long-Term Cognitive Effects Induced by Chronic Social Defeat During Early Adolescence

Four independent groups of mice (*n* = 7 per group) were exposed to 10 consecutive days of CSD or control manipulation during early adolescence from PND28 to 37. For short-term studies, two groups (one control and one defeated group) were evaluated in mid-adolescence in the Barnes Maze (BM) from PND39 to 50. The other two groups (one control and one defeated group) were group-housed without manipulation for 4 weeks until reaching early adulthood for the long-term studies. These mice were evaluated in the BM from PND67 to 78.

##### Experiment 4. Neuroendocrine Effects Induced by Chronic Social Defeat During Early Adolescence

For this experiment, 54 mice were used from PND25 to 65. One group (*n* = 3) was euthanized by decapitation to obtain blood from the trunk on PND25. The rest of the animals were exposed to 10 consecutive days of social defeat from PND28 to 37. Mice (groups of 3 animals) were euthanized by decapitation (to obtain trunk blood) at 5-day intervals, during or after the CSD from PND30 to 60. All blood samples were collected within 4 h of the dark cycle onset to control circadian variation in corticosterone. Blood samples were centrifuged at 14,500 rpm for 25 min at 4°C to obtain blood serum stored at −80°C until analysis. It is important to note that group-housed mice after social defeat were housed with mice of the same age and social experience to avoid the behavioral effects induced by social isolation.

## Results

### Experiment 1. Short- and Long-Term Depression-Like Behaviors Induced by Chronic Social Defeat During Early Adolescence

#### The Chronic Social Defeat Model During Early Adolescence Induced Enduring Depression-Like Behaviors

**FIGURE 1:**
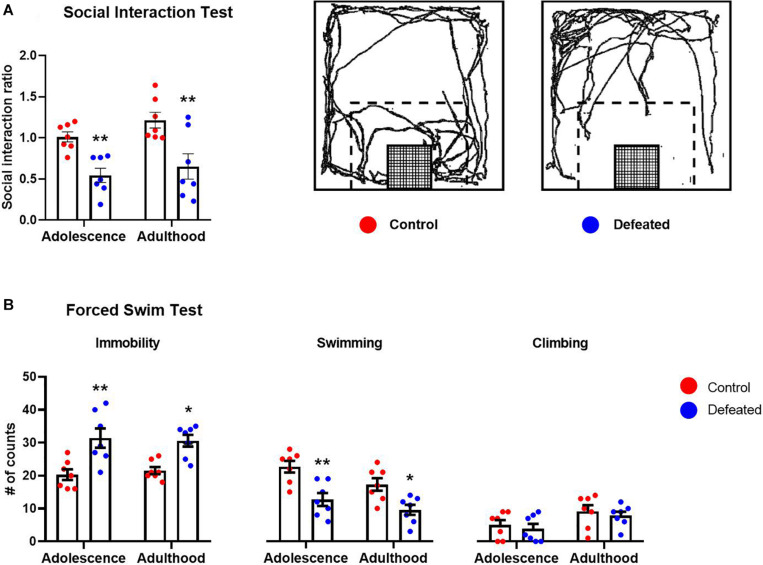
Effects of the CSD in the **(A)** social interaction test and **(B)** forced swimming test, in both adolescence and adulthood. Data are presented as the mean ± SEM of the social interaction ratio or the number of counts of the immobility, swimming, and climbing behaviors. Schematic representation of the social interaction arena illustrating the location and size of the interaction zone (dotted line) with respect to the wire-mesh enclosure (grid) in which a social target is placed. Representative track of the travel done by a control or defeated mouse when the social target was present **(A)**. Two-way ANOVA, *post hoc* Sidak’s test *p < 0.05, **p < 0.01, *n* = 7.

Figure 1A shows the effect of CSD during early adolescence on the social interaction ratio in the SIT in both adolescent and adult mice. We found a significant reduction in the social interaction ratio of defeated mice, in both adolescent (*p* = 0.008) and adult (*p* = 0.0014), compared to their respective control groups. The two-way ANOVA test yielded the following values, when analyzing the SIR: age [*F*(1, 12) = 2.17, *p* = 0.166], stress exposure [*F*(1, 12) = 24.15, *p* < 0.001], and the interaction between age and stress exposure [*F*(1, 12) = 0.206, *p* = 0.657].

Figure 1B shows the effect of the CSD during early adolescence on immobility, swimming, and climbing behaviors in the FST in both adolescent and adult mice. We found a significant increase in the immobility behavior in defeated mice, in both adolescents (*p* = 0.001) and adults (*p* = 0.007), compared to their respective control groups. The two-way ANOVA test yielded the following values, when analyzing the immobility behavior: age [*F*(1, 12) = 0.021, *p* = 0.886], stress exposure [*F*(1, 12) = 17.58, *p* = 0.001], and the interaction between age and stress exposure [*F*(1, 12) = 0.532, *p* = 0.479].

We found a significant decrease in the swimming behavior of defeated mice in both adolescents (*p* = 0.001) and adults (*p* = 0.011) compared to their respective control groups. The two-way ANOVA yielded the following values, when analyzing the swimming behavior: age[*F*(1, 12) = 12.31, *p* = 0.004], stress exposure [*F*(1, 12) = 15.97, *p* = 0.001], and the interaction between age and stress exposure [*F*(1, 12) = 0.875, *p* = 0.367].

Finally, in relation to climbing behavior, we did not find significant differences in the climbing behavior of defeated mice, in either adolescent (*p* = 0.843) or adult (*p* = 0.806) compared to their respective control groups. The two-way ANOVA test yielded the following values, when analyzing the climbing behavior: age [*F*(1, 12) = 32.06, *p* < 0.001], stress exposure [*F*(1, 12) = 0.348, *p* = 0.565], and the interaction between age and stress exposure [*F*(1, 12) = 0.009, *p* = 0.922].

### Experiment 2. Short- and Long-Term Effects on Serotonin Transporter Binding Induced by Chronic Social Defeat During Early Adolescence

#### The Chronic Social Defeat During Early Adolescence Induced Opposite and Age-Dependent Effects on Serotonin Transporter Binding in the Hippocampus

**FIGURE 2:**
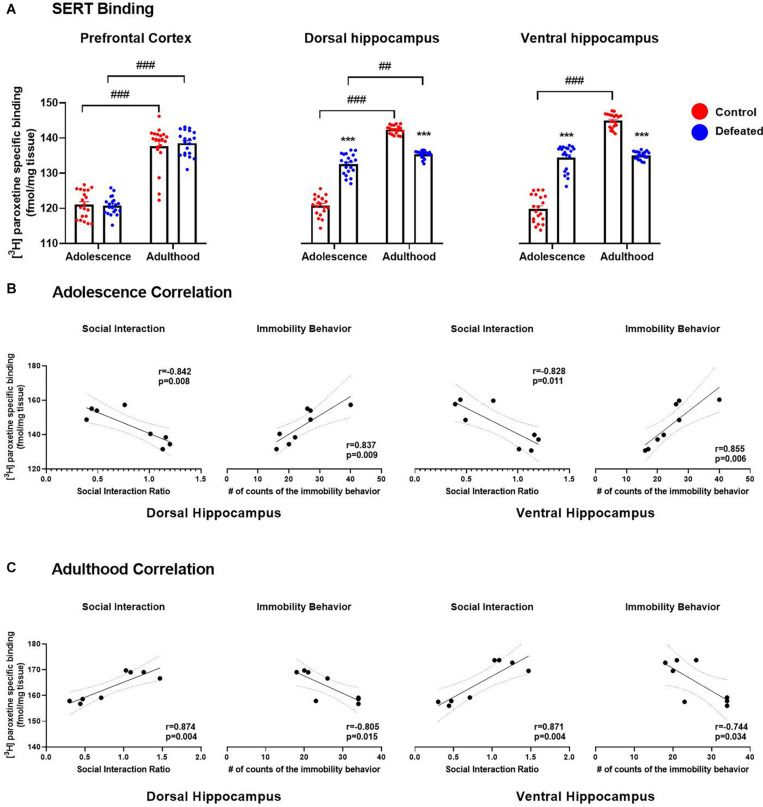
Effects of the CSD on SERT binding (fmol/mg tissue) in the prefrontal cortex, dorsal hippocampus, and ventral hippocampus in both adolescence and adulthood **(A)**. Data are presented as the mean ± SEM of the [^3^H] paroxetine specific binding (fmol/mg tissue). Two-way ANOVA, *post hoc* Tukey’s test ***p < 0.001 vs. control; ^###^*p* < 0.001, ^##^*p* < 0.01 vs. adulthood, *n* = 4. The color circles represent OD values (converted to fmol/mg tissue) for each area. Pearson correlation between SERT binding and social interaction ratio or immobility behavior in both dorsal and ventral hippocampus in adolescence **(B)** and adulthood **(C)**.

Figure 2A shows the effect of the CSD during early adolescence on SERT binding in the PFC and hippocampus (dorsal and ventral) in both adolescent and adult mice. In the PFC, we found a significant increase of SERT binding in adult mice, both control (*p* < 0.001) and defeated (*p* < 0.001), compared to adolescent mice. However, we did not find significant differences in SERT binding in the PFC of defeated mice, in either adolescent (*p* = 0.988) or adult (*p* = 0.843), compared to their respective control groups. A two-way ANOVA test yielded the following values, when analyzing SERT binding in the PFC: age [*F*(1, 19) = 272, *p* < 0.001], stress exposure [*F*(1, 19) = 0.068, *p* = 7.96], and the interaction between age and stress exposure [*F*(1, 19) = 0.644, *p* = 0.431].

In the dorsal hippocampus, we found a significant increase in SERT binding in adult mice, both control (*p* < 0.001) and defeated (*p* = 0.001), compared to adolescent mice. In adolescent mice, we found a significant increase in SERT binding in the dorsal hippocampus of defeated compared to its control group (*p* < 0.001). In contrast, in adult mice, we found a significant decrease in SERT binding in the dorsal hippocampus of defeated compared to its control group (*p* < 0.001). The two-way ANOVA test yielded the following values, when analyzing the SERT density in the dorsal hippocampus: age [*F*(1, 19) = 527.1, *p* < 0.001], stress exposure [*F*(1, 19) = 36.4, *p* < 0.001), and the interaction between age and stress exposure [*F*(1, 19) = 428.1, *p* < 0.001].

Similarly, in the ventral hippocampus, we found a significant increase in SERT binding in adult control mice compared to adolescent control mice (*p* < 0.001), but not in defeated mice. In adolescent mice, we found a significant increase in SERT binding in the ventral hippocampus of defeated mice compared to its control group (*p* < 0.001). On the other hand, in adult mice, we found a significant decrease in the SERT binding in the ventral hippocampus of defeated mice compared to its control group (*p* < 0.001). The two-way ANOVA test yielded the following values, when analyzing the SERT density in the ventral hippocampus: age [*F*(1, 19) = 389.5, *p* < 0.001], stress exposure [*F*(1, 19) = 14.42, *p* = 0.001], and the interaction between age and stress exposure [*F*(1, 19) = 848.9, *p* < 0.001].

#### Serotonin Transporter Binding in the Dorsal and Ventral Hippocampus Correlated With the Depression-Like Behaviors in an Age-Dependent Manner

Figure 2B shows the relation between SERT binding in the hippocampus (dorsal and ventral) and social interaction rate or immobility behavior in adolescence. In adolescent mice, the Pearson test revealed that SERT binding in the hippocampus (dorsal and ventral) was correlated negatively with the social interaction rate (dorsal: *r* = −0.842, *p* = 0.008; ventral: *r* = −0.828, *p* = 0.011), and positively with the immobility behavior (dorsal: *r* = 0.837, *p* = 0.009; ventral: *r* = 0.855, *p* = 0.006).

Figure 2C shows the relation between SERT binding in the hippocampus (dorsal and ventral) and social interaction rate or immobility behavior in adulthood. In adult mice, the Pearson test revealed that SERT binding in the hippocampus (dorsal and ventral) was correlated positively with the social interaction rate (dorsal: *r* = 0.874, *p* = 0.004; ventral: *r* = 0.871, *p* = 0.004), and negatively with the immobility behavior (dorsal: *r* = −0.805, *p* = 0.015; ventral: *r* = −0.744, *p* = 0.034).

Finally, the Pearson test did not reveal a correlation between SERT binding in the PFC and the social interaction ratio (adolescence: *r* = 0.301, *p* = 0.091; adulthood: *r* = 0.156, *p* = 0.711) or the immobility behavior (adolescence: *r* = 0.046, *p* = 0.913; adulthood: *r* = 0.197, *p* = 0.639), neither in mid-adolescence nor early adulthood (data no show).

### Experiment 3. Short- and Long-Term Cognitive Effects Induced by Chronic Social Defeat During Early Adolescence

#### The Chronic Social Defeat During Early Adolescence Impaired Long-Term Memory in the Mid-Adolescence and Impaired the Visuospatial Learning and Both Short- and Long-Term Memory in the Early Adulthood

Table 1 shows the effects of the CSD during early adolescence on the primary latencies in the acquisition phase of the Barnes maze in both adolescent and adulthood. In adolescence, we did not find significant differences in the primary latency in days 2, 3, and 4, compared to day 1, neither in control [*F*(3, 24) = 1.981, *p* = 0.143] nor defeated [*F*(3, 24) = 1.742, *p* = 0.185] mice. In adulthood, we found a significant reduction in the latency primary in days 2, 3, and 4 (*p* = 0.001), compared to day 1 in control mice [*F*(3, 24) = 15.65, *p* < 0.001]. However, in defeated mice, we did not find significant differences in the primary latency in days 2, 3, and 4, compared to day 1 [*F*(3, 24) = 0.912, *p* = 0.449].

**TABLE 1:** Effects of CSD in the acquisition phase of the Barnes maze in both adolescence and adulthood.

		**Day 1**	**Day 2**	**Day 3**	**Day 4**
**Adolescence**	Control	62.0 ± 12.2	34.4 ± 10.6	76.4 ± 16.2	59.4 ± 9.3
	Defeated	78.2 ± 19.4	68.7 ± 15.6	109.6 ± 18.2	58.3 ± 12.8
**Adulthood**	Control	55.0 ± 6.8	19.3 ± 3.3**	20.9 ± 2.4**	23.7 ± 2.8*
	Defeated	48.7 ± 10.6	31.5 ± 5.2	51.0 ± 7.4	44.9 ± 11.4

Data are presented as the mean ± SEM of the primary latency. RM—ANOVA, post hoc Dunnett’s test, **p < 0.01, *p < 0.05 vs. its respective day 1 for each group.

**FIGURE 3:**
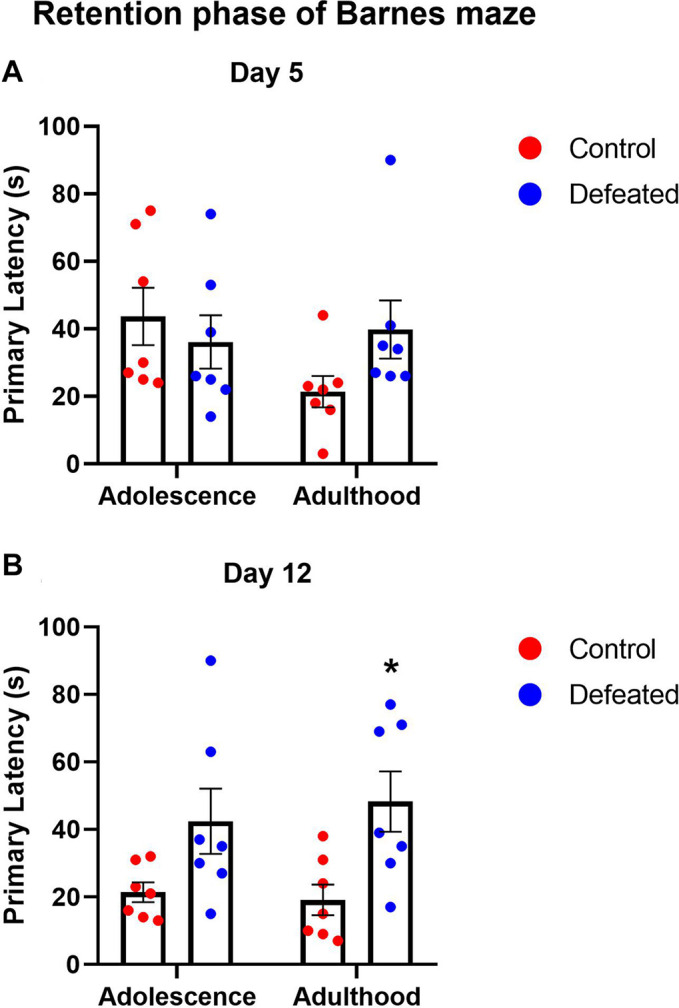
Effects of the CSD in the Barnes maze on the primary latency after 5 days **(A)** and 12 days **(B)** of the retention phase in both adolescence and adulthood. Data are presented as the mean ± SEM of the primary latency. Two-way ANOVA, *post hoc* Sidak’s test, *p < 0.05, *n* = 7.

Figures 3A,B show the effect of the CSD during early adolescence on the primary latency in day 5 or 12, respectively, of the retention phase of the BM in both adolescence and adulthood. On day 5, we did not find significant differences in the primary latency of defeated mice, neither adolescent (*p* = 0.737) nor adults (*p* = 0.188). The two-way ANOVA test yielded the following values: age [*F*(1, 12) = 1.295, *p* = 0.277], stress exposure [*F*(1, 12) = 0.602, *p* = 0.452], and their interaction age X stress exposure [*F*(1, 12) = 2.538, *p* = 0.137]. On day 12, we found a significant increase in the primary latency in adults (*p* = 0.015), but not in adolescent (*p* = 0.092). The two-way ANOVA test yielded the following values: age [*F*(1, 12) = 0.059, *p* = 0.812], stress exposure [*F*(1, 12) = 13.34, *p* = 0.003], and the interaction between age and stress exposure [*F*(1, 12) = 0.306, *p* = 0.589].

### Experiment 4. Neuroendocrine Effects Induced by Chronic Social Defeat During Early Adolescence

#### The Chronic Social Defeat During Early Adolescence Induced an Increase in Serum Corticosterone Content in Mid-Adolescence and Delayed Its Return to Basal Levels in Early Adulthood

**FIGURE 4:**
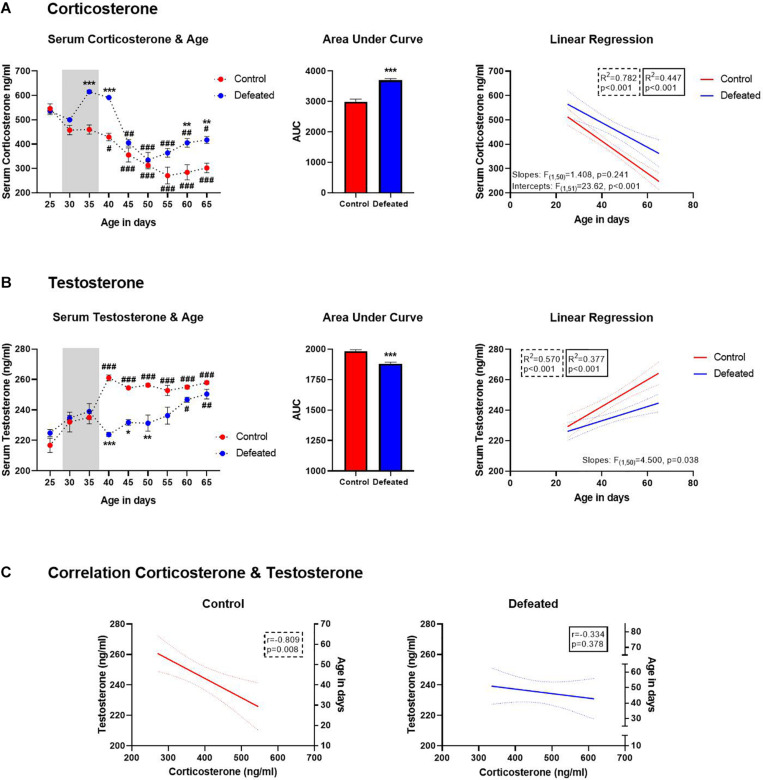
**(A)** Serum corticosterone content (ng/ml) before, during, and after of the CSD (gray bar), data are presented as the mean ± SEM of the serum corticosterone content, two-way ANOVA *post hoc* Tukey’s test, **p < 0.01, ***p < 0.01 vs. control, ^#^*p* < 0.05, ^##^*p* < 0.01, ^###^*p* < 0.001 vs. day 25 in both groups (left side). Area under the curve for serum corticosterone before, during, and after of the CSD (central side). Serum corticosterone was linearly correlated with the age of mice in both control and defeated group (right side). **(B)** Serum testosterone content (ng/ml) before, during, and after of the CSD (gray bar), data are presented as the mean ± SEM of the serum testosterone content, Two-way ANOVA *post hoc* Tukey’s test, *p < 0.05, **p < 0.01 ***p < 0.01 vs. control,^ #^*p* < 0.05, ^##^*p* < 0.01, ^###^*p* < 0.001 vs. day 25 in both groups. Area under the curve for serum testosterone before, during, and after of the CSD (central side). Serum testosterone was linearly correlated with the age of mice in both control and defeated group (right side). **(C)** Left side, in control mice the serum testosterone was linearly correlated with the serum corticosterone; right side, in defeated mice there was no correlation between serum testosterone and corticosterone.

Figure 4A shows serum corticosterone content before, during, and after the CSD, the area under the curve (AUC), and the linear regression between serum corticosterone content and the age of mice (at 5 days intervals). We observed a significant decrease in serum corticosterone content from PND40 to 65 in control mice, and from PND45 to 65 for defeated mice, in both cases compared to their respective value on PND25. However, defeated mice showed a significant increase in serum corticosterone content in the PND35 (*p* < 0.001), 40 (*p* < 0.001), 60 (*p* = 0.009), and 65 (*p* = 0.014) compared to the respective day in control mice. The two-way ANOVA test yielded the following values when analyzing serum corticosterone content before, during, and after the social defeat: age of mice [*F*(8, 16) = 54.46, *p* < 0.001], stress exposure [*F*(1, 2) = 77.41, *p* = 0.012], and their interaction age of mice X stress exposure [*F*(8, 16) = 6.258, *p* < 0.001].

We found a significant increase in the AUC of defeated mice [*t*(46) = 7.039, *p* < 0.001] compared to the control group. Also, corticosterone content was linearly correlated with the age of mice in both control [*F*(1, 25) = 89.87, *p* < 0.001, *R*^2^ = 0.782] and defeated [*F*(1, 25) = 20.26, *p* < 0.001; *R*^2^ = 0.447] mice. Older mice, both control and defeated, tend to have the lowest serum corticosterone content. Although the differences between control and defeated slopes were not significant [*F*(1, 50) = 1.408, *p* = 0.241], the differences between their intercepts were extremely significant [*F*(1, 51) = 23.62, *p* < 0.001]. So, we used the equation obtained from linear regression for defeated mice (y = −5.065^∗^x+691) to determine the age at which defeated mice would reach serum corticosterone content observed in the control group at PND65 (302.1 ± 19.15 ng/ml) and estimated that defeated mice would reach this corticosterone content in the PND 73 ± 7.

#### The Chronic Social Defeat During Early Adolescence Induced a Decrease in Serum Testosterone Content From Mid-Adolescence to Early Adulthood

Figure 4B shows serum testosterone content before, during, and after the CSD, the AUC, and the linear regression between serum testosterone content and the age of mice (at 5 days intervals). As expected, an increase of testosterone content was observed in control mice from PND40 to 65, when compared to PND25. In contrast, in defeated mice, testosterone content increase was observed until PND60 (*p* = 0.021) and 65 (*p* = 0.004), when compared to PND25. Moreover, defeated mice showed a significant decrease in serum testosterone content on PND40 (*p* < 0.001), 45 (*p* = 0.014), and 50 (*p* = 0.041) compared to control mice at the same days. The two-way ANOVA test yielded the following values when analyzing serum testosterone content before, during, and after social defeat: age of mice [*F*(8, 16) = 14.68, *p* < 0.001], stress exposure [*F*(1, 2) = 37.39, *p* = 0.02], and their interaction age of mice X stress exposure [*F*(8, 16) = 10.10, *p* < 0.001].

Also, we found a significant decrease in the AUC of defeated mice[*t*(46) = 5.822, *p* < 0.001] compared to the control group. Serum testosterone content was linearly correlated with the age of mice in both control [*F*(1, 25) = 33.2, *p* < 0.001, *R*^2^ = 0.570] and defeated [*F*(1, 25) = 15.13, *p* < 0.001; *R*^2^ = 0.377] mice; being higher in older mice under both conditions. The differences between control and defeated slopes were significant [*F*(1, 50) = 4.500, *p* = 0.038]. Therefore, we used the equation obtained from linear regression for defeated mice (y = 0.4677^∗^x+214) to determine the age at which they would reach serum testosterone content observed in the control group at PND65 (258 ± 1.019 ng/ml) and estimated that defeated mice would reach this corticosterone content in the PND94 ± 3.

#### The Chronic Social Defeat During Early Adolescence Reverted the Negative Correlation Between Serum Testosterone and Corticosterone Content Observed in Control Mice

Figure 4C shows the relation between serum testosterone and corticosterone content in both control and defeated mice, respectively. In control mice, the Pearson test revealed that serum testosterone content was negatively correlated with serum corticosterone content (*r* = −0.792, *p* = 0.019), while in defeated mice, the Pearson test did not reveal any correlation between testosterone and corticosterone (*r* = −0.329, *p* = 0.425).

## Discussion

Our results show that exposure to CSD during early adolescence induces depression-like behaviors in mid-adolescence and early adulthood. Also, this manipulation induces an impairment of long-term memory in mid-adolescence; and deficiencies in learning and memory in early adulthood. Interestingly, CSD during early adolescence produces long-lasting neuroendocrine alterations and opposite and age-dependent effects in SERT binding in the hippocampus (dorsal and ventral) that correlated with depression-like behaviors.

In line with our results, it has been shown that CSD during adolescence induced social avoidance 24 h and up to 2, 4, and 6 weeks after stress cessation (Huang et al., 2013; Iñiguez et al., 2014, 2016; Zhang et al., 2016; Mouri et al., 2018; Hasegawa et al., 2019). However, those studies employed mice of different strains as adolescents and aggressors, and the social target was a mouse of the aggressor strain. Therefore, it is difficult to interpret whether social avoidance behavior induced by CSD was a specific behavior toward the phenotypic characteristics of the aggressor or a generalized behavior toward any social target regardless of its characteristics. Here, we ran the social defeat only with SW mice (as adolescents, aggressors, and social targets) and showed that adolescent SW mice defeated by an aggressor of the same strain developed social avoidance toward another mouse of the same age and strain, both 24 h (mid-adolescence) and 4 weeks (early adulthood) after stress cessation. Overall, these results show that social avoidance behavior induced by CSD during adolescence is a long-lasting generalized behavior toward any social target and is not dependent on the social target phenotypic characteristics. Also, it highlights the age of exposure (early adolescence) to social defeat as a relevant factor for the persistence of social avoidance behavior.

Regarding behavioral despair induced by CSD, results are contradictory and age-dependent. Adult and adolescent C57BL/6 defeated mice did not display behavioral despair in the FST nor the tail suspension test (TST) 24 h after social defeat (Krishnan et al., 2007; Mouri et al., 2018). Conversely, adolescent C57BL/6J defeated mice displayed behavioral despair in both the FST (Huang et al., 2013; Iñiguez et al., 2014; Iñiguez et al., 2016) and TST (Shimizu et al., 2020), but only 24 h after social defeat. To our knowledge, this is the first study that reports that defeated adolescent SW mice displayed long-lasting behavioral despair since it was present 24 h (mid-adolescence) and 4 weeks (early adulthood) after stress cessation. These results suggest that in addition to the age of stress exposure, the mice strain should be taken into consideration at the time to evaluate the long-lasting depression-like behaviors induced by social defeat.

On the other hand, in control mice, we found an age-dependent increase in SERT binding in both the PFC and hippocampus (dorsal and ventral) since adults exhibited higher SERT binding than adolescent mice. In line with our results, by quantitative autoradiography assay, Mitchell et al. (2016) reported that adult mice show higher SERT binding in the hippocampus and amygdala than adolescent mice, and comparable results have been reported in the PFC, cingulate cortex, and raphe nuclei from rats (Moll et al., 2000; Bouet et al., 2012). Interestingly, by positron emission tomography (PET) in humans, Selvaraj et al. (2011) found a positive correlation between age (mean 41.9 ± 11.4 years) and the SERT binding in the amygdala, frontal cortex, insula, and putamen from healthy male controls. In this case, older healthy controls had higher SERT binding (Selvaraj et al., 2011). Despite the fact that the ontogeny of SERT during the transition from adolescence to adulthood has been poorly studied, the evidence suggests an age-dependent increase in SERT binding in the serotonergic projection areas such as the PFC, hippocampus, and amygdala, which is consistent with our results.

In mid-adolescence, adolescent defeated mice showed a higher SERT binding in both the DH and VH than control mice. It has been reported that 24 h after CSD, adult defeated rodents showed higher levels of SERT mRNA and SERT protein than controls in the raphe nuclei and its projecting regions such as the hippocampus, frontal cortex, and amygdala (Filipenko et al., 2002; Zhang et al., 2012, 2017). Moreover, it has been reported that a single social defeat in rodents increases both the hippocampal 5-HT levels (Keeney et al., 2006) and c-fos expression in the DRN (Cooper et al., 2009), suggesting hyperactivity of the serotonergic system in response to perceived aggression (Hammels et al., 2015). Therefore, it has been proposed that social defeat-induced serotonergic hyperactivity could trigger feedback mechanisms responsible for the normalization of its activity, one of which could be the SERT up-regulation (Filipenko et al., 2002; Zhang et al., 2012, 2017). Future studies could provide further support to this proposal.

In early adulthood, adolescent defeated mice showed a lower SERT binding in both the DH and VH than control mice. Although no works have used an experimental approach like ours, our results are comparable and consistent with previous preclinical reports in which adult mice were used. It has been reported that CSD, unpredictable chronic mild stress, and chronic corticosterone treatment produce low levels of SERT mRNA in the raphe nuclei (Boyarskikh et al., 2013), low levels of SERT protein in the hippocampus (Tang et al., 2013, 2014), and a low SERT binding in the hippocampus, striatum, thalamus, and cortex (Reisinger et al., 2019). In humans, the reduction of SERT binding in the brain has been suggested as a putative biomarker of depression (Selvaraj et al., 2011; Newberg et al., 2012) since several neuroimaging studies have shown a reduction in SERT binding in different brain areas from drug-naïve depressed patients (Malison et al., 1998; Parsey et al., 2006; Joensuu et al., 2007; Selvaraj et al., 2011; Newberg et al., 2012). Our results are in line with both preclinical and clinical studies. We found that CSD during adolescence alters the normal development of SERT expression since we observed stress-induced SERT upregulation in mid-adolescence, which could be interpreted as a response to the perceived aggression. In contrast, we found long-lasting stress-induced SERT downregulation in early adulthood, which could be interpreted as a premature maturation that did not reach normal adulthood development.

To our knowledge, this is the first work that evaluates the development of depressive endophenotypes and changes of SERT binding in the brain induced by CSD during adolescence. We decided to evaluate SERT binding in both DH and VH since it has been described that DH performs primarily cognitive functions, and VH relates to stress, emotion, and affect (Fanselow and Dong, 2010). In mid-adolescence, there was a correlation between depression-like behaviors and SERT binding in the DH and VH. Thus, mice with higher scores of depression-like behaviors (social interaction ratio and counts of immobility) present higher SERT binding. In support of these results, higher levels of SERT protein in the DRN, hippocampus, frontal cortex, and amygdala of adult defeated rats were associated with anhedonia (Zhang et al., 2012).

On the other hand, there was also a correlation between depression-like behaviors and SERT binding in the DH and VH in early adulthood. In this case, mice with higher scores of depression-like behaviors presented lower SERT binding. Similar to our results, lower SERT protein levels in the hippocampus, induced by unpredictable chronic mild stress and chronic corticosterone treatment in adult rats, were related to anhedonia and behavioral despair (Tang et al., 2013, 2014). Interestingly, a neuroimaging study reported a negative correlation between symptomatology from acutely depressed patients and SERT binding levels in the frontal cortex (Selvaraj et al., 2011). Overall, these results from preclinical and clinical studies highlight that stress-induced and age-dependent changes in the hippocampal SERT expression are intimately related to the development and persistence of depression-like behaviors.

However, we did not find significant differences in SERT binding in the PFC between control and defeated mice, neither in mid-adolescence nor early adulthood. Although our results are in line with previous reports in drug-naïve depressed children and adolescents (Dahlström et al., 2000), they are different from those reported in both drug-naïve depressed adults and adult defeated rodents, in which low and high levels of SERT binding in the frontal cortex were found, respectively (Selvaraj et al., 2011; Zhang et al., 2012). In rodents, we think that the age at which the stressor was administered, early adolescence vs. adulthood, could be responsible for this discrepancy. Future studies should investigate the ontogeny of SERT during the transition from adolescence to adulthood in the PFC.

Impairments in learning and memory are frequently cognitive deficits observed in depressed people (Bourke et al., 2012). Thus, we decide to investigate this cognitive domain in mice defeated during early adolescence and found that social defeat in this stage induced long-term memory impairment in the BM in mid-adolescence. In line with our results, rats stressed during early adolescence showed short-term memory impairment in the BM (Kim et al., 2017) and visuospatial learning and short-term memory impairments in the Morris maze (Jianhua et al., 2017) in late adolescence. Conversely, rats stressed during early adolescence did not show cognitive impairments in the Morris maze in late adolescence (Isgor et al., 2004). We think that performance of adolescent rodents in visuospatial learning and memory could be influenced by age, and this could explain the contradictory results observed in both the BM and Morris maze so, future studies should continue to focus on this issue.

We also found that social defeat during early adolescence induced visuospatial learning and short- and long-term memory impairments in early adulthood. Like our results, adolescent rats exposed to physical stress in early adolescence (Isgor et al., 2004) and restriction stress in mid-adolescence (Trofimiuk et al., 2019) showed visuospatial learning and short-term memory impairments in the Morris maze and short-term memory impairment in the BM, respectively, in adulthood. Moreover, chronic corticosterone treatment induced visuospatial learning and short- and long-term memory impairments in the BM in adult rats (McLay et al., 1998; Darcet et al., 2014). Although the BM and the Morris maze are different in design, procedure, and stressful experience, both evaluate the hippocampal-based visuospatial learning and memory. Thus, these results show that chronic exposure to stress or stress-related hormones, either in adolescence or adulthood, have detrimental effects on visuospatial learning and memory.

In rodents, the HPA axis hyperactivity induced by chronic stress leads to adverse effects on cognitive processes. Also. in depressed people, the enhanced HPA axis activity induces adverse effects on cognitive performance (Darcet et al., 2014). In the present study, we found that defeated mice showed higher serum corticosterone levels in mid-adolescence and early adulthood just when the cognitive impairments were observed. The adolescent hippocampus is sensitive to chronic stress and stress-related hormones (Romeo, 2016) since chronic stress reduces the pyramidal dendritic branching in the hippocampus of adolescent male and female rats (Eiland and Romeo, 2013) and slows the normal development of the hippocampal pyramidal and granular cell layers during the transition from adolescence to early adulthood in male rats (Isgor et al., 2004). In addition, one neuroanatomical finding reported in depressed people is the decreased volume of the hippocampus, which is associated with the length of depression (Bremner et al., 2000; Sheline et al., 2003). Although we did not evaluate the hippocampal morphology, we observed the most significant changes in SERT binding in this area. Thus, it is possible that anatomical and functional changes induced by chronic stress in the hippocampus could be responsible for the long-lasting impaired cognitive performance observed in our defeated mice. Further studies are needed to confirm this assumption.

In our work, CSD during early adolescence induced a decreased serum testosterone content from mid-adolescence to early adulthood. It has been described that adult male rats showed low levels of plasma testosterone after losing a physical confrontation (Schuurman, 1980; Blanchard et al., 1993) and, interestingly, socially anxious men showed a pronounced drop in testosterone levels after losing a competition (Maner et al., 2008). In line with our results, male golden hamsters exposed to CSD during early adolescence showed reduced levels of plasma testosterone in mid-adolescence (Wommack et al., 2004) and, male rats exposed to CSD during adulthood also showed low levels of plasma testosterone (Huhman et al., 1991; Rygula et al., 2006; Buwalda et al., 2010). However, only Rygula et al. (2006) found a parallel development of anhedonia. In humans, low testosterone levels are associated with depression, which is evident in men with hypogonadism, a condition in which reduced functional activity of the gonads results in decreased testosterone levels (McHenry et al., 2014; Zitzmann, 2020). Moreover, like hypogonadal men, rodents with low testosterone levels can exhibit increased depressive-like behaviors such as behavioral despair in the FTS and anhedonia in the sucrose preference test (McHenry et al., 2014). We showed that CSD during early adolescence reduced serum testosterone levels from mid-adolescence to early adulthood and developed long-lasting depression-like behaviors.

We observed a negative correlation between serum testosterone and corticosterone levels in the transition from early adolescence to early adulthood in control mice. However, in defeated mice, this correlation was absent. In physiological conditions, from early life to adulthood, gonadal hormones can differentially affect the HPA axis activity and, therefore, its future response to stress, resulting in sex differences in the responsivity of this axis in adulthood (Oyola and Handa, 2017). It is known that the general pattern of the HPA axis response to stress is characterized by a decline in stress reactivity as animals progress through adolescent development (Romeo, 2010). McHenry et al. (2014) proposed that in males, the levels of testosterone and the corticotrophin release hormone (CRH), the initiator hormone of the stress response, could be inversely related, and our results support this proposal.

Moreover, we found that serum corticosterone levels, from early adolescence to early adulthood, in both control and defeated mice were negatively correlated with the age of mice. In this case, older mice had lower corticosterone levels. However, defeated mice showed higher serum corticosterone levels than control mice in two moments, in mid-adolescence (PND35, 40) and early adulthood (PND60). In mid-adolescence, elevated serum corticosterone levels, attributable to the HPA axis response to the CSD, could be responsible for the reduced levels of serum testosterone observed in defeated mice (from PND40 to 60). In support of this proposal, the CUMS induced elevated levels of plasma corticosterone and, at the same time, reduced plasma testosterone levels and testicular dysfunction in male rats (Lu et al., 2015; Sakr et al., 2015). It is known that stress and stress-hormones can inhibit the release of the gonadotrophin release hormone (GnRH) from the hypothalamus, and glucocorticoids can inhibit the release of luteinizing hormone (LH) from the anterior pituitary as well as the synthesis of testosterone through reductions in the number of Leydig cells because of apoptosis (Hardy et al., 2005; Toufexis et al., 2014). These mechanisms could explain the reduced levels of testosterone observed in defeated mice and the reduced levels of testosterone which could be responsible for the elevated levels of serum corticosterone observed in defeated mice in early adulthood. In basal conditions and under stress-induced HPA axis activity, males typically show lower ACTH and glucocorticoid levels than females, and these sex differences in HPA axis function are attributed to inhibitory effects of testosterone in males (Viau, 2002). The zona fasciculata from the adrenal cortex (responsible for the synthesis and release of glucocorticoids) decreases in its growth rate in males as a direct result of testosterone production during adolescence (Bale and Epperson, 2015). In the brain, testosterone can regulate HPA axis activity in rats and humans through its respective androgen receptor (AR), which acts directly on the CRH gene promoter and inhibits its transcription (Bao et al., 2006; Lu et al., 2015). These results highlight the anti-stress effect of testosterone which becomes more evident between both sexes during adolescence. We think that CSD during early adolescence leads to the loss of this anti-stress effect and alters the HPA axis response to stressors during adulthood. Moreover, other long-term consequences of CSD during early adolescence, such as reduced SERT expression observed in the hippocampus of defeated mice, could contribute to this altered HPA axis response since mice with reduced or absent SERT showed an increased sensitivity to stress because of altered negative feedback (Jiang et al., 2009). These results show the complex interaction between the HPA and the HPG axes during adolescence and highlight the long-lasting neuroendocrine and behavioral consequences of its inadequate interaction because of chronic stress exposure in early adolescence.

Overall, these results show that CSD during early adolescence induces long-lasting depression-like behaviors. They also reveal the critical and age-dependent role of SERT in the normal brain development in the transition from adolescence to adulthood and the pathological development of depression-like behaviors. Also, this study highlights the vulnerability of the interaction between HPA and HPG axes in early adolescence. Furthermore, all these alterations observed in defeated mice were presented analogously to the clinical evolution observed in young people with depression. Thus, our results add more construct and face validity to the CDS model in adolescent male mice, which can be used to investigate the neurobiology process underlying the stress-induced depression in young people and the screening for new compounds useful for the treatment of depression in this age group.

## Data Availability Statement

The raw data supporting the conclusions of this article will be made available by the authors, without undue reservation.

## Ethics Statement

The animal study was reviewed and approved by the Comité Interno para el Cuidado y Uso de los Animales de Laboratorio (CICUAL) del Cinvestav.

## Author Contributions

HM-G did the experimental work, statistical analysis, wrote the manuscript, and participated in the conception and design of the experiments. LM-N participated in the conception and design of SERT experiments. EL-P participated in the conception and design of the neuroendocrine experiments. CL-R and EE-C participated in the conception and design of all experiments and in the review process. All authors approved the final version of this manuscript.

## Conflict of Interest

The authors declare that the research was conducted in the absence of any commercial or financial relationships that could be construed as a potential conflict of interest.

## Publisher’s Note

All claims expressed in this article are solely those of the authors and do not necessarily represent those of their affiliated organizations, or those of the publisher, the editors and the reviewers. Any product that may be evaluated in this article, or claim that may be made by its manufacturer, is not guaranteed or endorsed by the publisher.
